# The interaction between general or abdominal obesity and hypertension on the risk of type 2 diabetes mellitus: a cross-sectional analysis in Iranian adults from the RaNCD cohort study

**DOI:** 10.1186/s12889-024-18290-7

**Published:** 2024-03-11

**Authors:** Yahya Pasdar, Shahab Rezaeian, Ehsan Mohammadi, Fatemeh Khosravi shadmani, Narges Shahnazi, Farid Najafi, Mehdi Moradi Nazar, Mitra Darbandi

**Affiliations:** 1 Research Center for Environmental Determinants of Health (RCEDH), Health Institute, Kermanshah University of Medical Sciences, Kermanshah, Iran; 2 Infectious Disease Research Center, Health Institute, Kermanshah University of Medical Sciences, Kermanshah, Iran; 3 Kermanshah University of Medical Sciences, Kermanshah, Iran; 4 Cardiovascular Research Center, Kermanshah University of Medical Sciences, Kermanshah, Iran

**Keywords:** Abdominal obesity, General obesity, Hypertension, Diabetes mellitus

## Abstract

**Background:**

Interactions between risk factors may influence disease severity. Knowing this relationship is important for preventive interventions and disease control. The purpose of this study was to determine the interactions effects of obesity and hypertension on the risk of type 2 diabetes mellitus (T2DM).

**Methods:**

The data of 9,283 adults 35 to 65 years were examined from the cohort study of Ravansar Non-Communicable Disease (RaNCD). Waist circumference (WC) was used to identify both general and abdominal obesity based on body mass index (BMI). To assess the interaction between hypertension and obesity (general/abdominal) and the risk of T2DM, the additive interaction was calculated.

**Results:**

The adjusted odds ratios for T2DM were 2.38 (1.67, 3.41) in men and 4.02 (2.47, 6.47) in women for the combinations of hypertension and abdominal obesity. The adjusted odds ratios for T2DM were 2.53 (1.63, 3.82) in men and 2.66 (1.92, 3.70) in women for the combinations of hypertension and general obesity. The results of the additive interaction indicators were inconsistent with gender. The relative excess risk due to interaction (interaction between hypertension and central obesity) (RERI), attributable proportion due to interaction (AP) and synergy index (SI) were0.27 (-1.01, 1.54), 0.11 (-0.41, 0.63) and 1.23 (0.41, 3.68) in male and were 0.61 (-1.12, 2.33), 0.23 (0.08, 0.37) and 1.26 (0.60, 2.61) in female, respectively.

**Conclusion:**

General/abdominal obesity and hypertension have a synergistic effect on the risk of T2DM. The recommendation for preventing T2DM is lifestyle modification. Large longitudinal studies are necessary to investigate causal relationships.

## Introduction

According to the World Health Organization (WHO), non-communicable diseases (NCDs) are the leading cause of death at global level [1]. Type 2 diabetes Mellitus (T2DM) *is* one of the most important NCDs responsible for 2.74% of all deaths globally [2]. Iran follows the same pattern, where 3% of all deaths were associated with T2DM in Iran [2]. The age standardized mortality rate for T2DM in Iran has shown a steady trend since 2015 and is expected to increase slightly by 2030 [3].

T2DM is a complex multifactorial disease associated with various risk factors [4, 5]. Scientific evidence has shown that hypertension and T2DM occur together, and the risk of developing T2DM is higher in hypertensive patients than in non-hypertensive [6, 7]. Generally, there is a positive correlation between impaired glucose tolerance and high blood pressure; and hypertension is reported in more than two-thirds of patients with T2DM [7, 8]. In addition, obesity as a major health problem significantly increases the risk of NCDs such as T2DM [9, 10]. Recently, in the study of Hu et al., it was shown that the risk of T2DM in hypertensive and obese individuals was significantly higher compared to those with a normal weight and without hypertension [9]. This relationship indicates that the combined effect of two risk factors together is greater than their effect alone (synergistic interactions). The impact of central obesity on diabetes risk is more significant than that of general obesity [11]. After puberty, fat deposition shifts to the visceral location in males, and in women, this shift occurs at menopause. Additionally, individuals with a normal BMI but a high waist-to-hip ratio (WHR) have a threefold increase in the rate of diabetes [12]. Reducing waist circumference (WC) may lower the risk of developing type 2 diabetes [13]. Our study considers both general and abdominal obesity to account for the complexity of obesity as a condition with diverse manifestations. This comprehensive approach allows for a detailed exploration of the obesity-T2DM relationship and provides a holistic understanding of how different aspects of obesity may interact and influence the risk of Type 2 Diabetes Mellitus in our specific study population.

According to the mentioned facts, the results of association between diabetes and obesity or hypertension should not be explained alone; rather it is essential to consider the interactions happening among them. The interactions between the risk factors may affect the severity of the condition. The study was conducted on 10,000 adults who participated in the first phase of the Ravansar Non-Communicable Disease (RaNCD) cohort study. It is important to note that obesity and overweight are relatively common in this population, especially among women [14], which emphasizes the significance of the present study. Consequently, this research was done to assess the effects of interactions between general or abdominal obesity and hypertension on the risk of T2DM in adults in western Iran.

## Method

### Participants

A cross-sectional analysis of data from the RaNCD cohort study was conducted in this study. The RaNCD cohort study is part of the prospective epidemiologic studies of the Iranian Adult Cohort (PERSIAN) (https://persiancohort.com/), which examines 10,000 adults (35 to 65 years old). The RaNCD study’s baseline phase was performed out between November 2014 and February 2017, the protocol of which has been previously published [14]. Pregnant women (*n* = 138), participants with cancer (*n* = 80), people with a body mass index (BMI) less than 18.5 kg/m2 (*n* = 173), women with gestational diabetes (*n* = 217) and missing data (*n* = 156) were excluded from the study. Finally, 9,283 people were examined.

### Data Collection

Sociodemographic information was performed by educated experts of the RaNCD cohort center in person using digital questionnaires. The RaNCD study protocol states that, current smokers were defined as those who smoked 100 or more cigarettes annually. Drinking 200 ml or less of alcohol or 45 ml or more of liquor once a week for at least six months was considered to be consuming alcohol. Socio-economic status (SES) was established using principal component analysis (PCA) using level of education, place of residence, housing and amenities [15]. Then, they were divided into three categories, ranking them from lowest to highest. The PERSIAN cohort questionnaire was used to measure the participants’ physical activity levels, after which the individuals were divided into three groups according on their levels (low: 24-36.5, moderate: 36.6–44.4, and vigorous: 36.6–44.4 inches MET/hour per day). Height of participants using a stadiometer (BSM 370, Biospace Inc); The waist circumference (WC), visceral fat area (VFA), and body mass index (BMI) were assessed using a bio-impedance BIA analyzer (Inbody 770, Inbody Co, Seoul, Korea). The cohort research protocol provides specifics on the data gathering methodology [14].

### Definitions

Hypertension is defined as having a systolic blood pressure (SBP) more than 140 mmHg, a diastolic blood pressure (DBP) below 90 mmHg, or using antihypertensive medications. Diabetic patients were defined as those with FBS 126 mg/dL and/or a history of using medication to treat T2DM. General obesity has been defined as a BMI 30 kg/m2 [14]. According to the National Cholesterol Education Program (NCEP) guidelines, WC was used to characterize abdominal obesity. Therefore, abdominal obesity has been described as WC > 88 cm for females and > 102 cm for males [16].

### Statistical analysis

Stata 14.2 (Stata Corp, College Station, Texas) software was used to analyze the data. To descriptive reports, mean ± standard deviation (SD) and number (percentage) were used. Chi-square tests and one-way ANOVA were used to compare the baseline characteristics of the participants. To investigate how general/abdominal obesity and hypertension affect the risk of T2DM, a multivariate logistic regression analysis was conducted. *P* value < 0.05 indicated a statistically significant.

Additive interaction analysis between general obesity, abdominal obesity and hypertension on the developing of T2DM was performed utilizing the Excel table Anderson created [14]. The relative excess risk due to interaction (RERI) which is calculated as: RERI = OR11 - OR10-OR01 + 1, the attributable proportion due to interaction (AP) which is calculated as: AP = RERI/OR11, and the synergy index (SI) which is calculated as: SI = (OR11–1)/ [(OR10–1) + (OR01– 1)] were calculated using the Excel Table [17]. Multiplicative interaction was evaluated using the ratio of ORs: OR11/ (OR10 × OR01). If the 95% confidence interval (CI) of RERI and AP include 0 or the 95% CI of SI contain 1, the additive interaction would be considered no statistical significance [18].

## Results

### Baseline characteristics

A total of 9,283 participants were included in the study (men: 4,545 and women: 4,738). The average age of the participants was 47.43 years. The proportion of T2DM in the studied population was 8.38% (8.16% men and 8.59% women). The proportion of hypertension, general and abdominal obesity were 16.03%, 26.64% and 55.22%, respectively. There were significant differences in the proportion of hypertension between the Non-T2DM and T2DM groups (14.50% vs. 32.78%; *P* < 0.001). The proportion of general obesity in the T2DM group (25.70%) was significantly higher than the Non- T2DM group (36.89%), (*P* < 0.001). The proportion of abdominal obesity between Non- T2DM and T2DM group had significant differences (54.10% vs. 67.48%, *P* < 0.001) **(**Table 1).

**Table 1:** Baseline characteristics between study participants with and without type 2 diabetes mellitus

Variables	Non-T2DM group (*n* = 8,505)	T2DM group (*n* = 778)	*P* value
Age (year)	47.02 ± 8.21	52.01 ± 7.34	< 0.001
Gender, n (%)			
Men	4174 (91.84)	371 (8.16)	0.458
Women	4331 (91.41)	407 (8.59)	
Residency, n (%)			
Urban	5064 (90.82)	512 (9.18)	0.001
Rural	3441 (92.82)	266 (7.18)	
Socioeconomic status, n (%)			
1(lowest)	2777 (32.66)	242 (31.15)	0.242
2	2822 (33.19)	281 (36.16)	
3(Highest)	2904 (34.15)	254 (32.69)	
Current smoker, n (%)	981 (11.60)	84 (10.81)	< 0.001
Alcohol drinking, n (%)	427 (5.02)	34 (4.37)	0.424
Physical activity (Met h/day),n (%)			
Low (24-36.5)	2548 (29.96)	285 (36.63)	< 0.001
Moderate (36.6–44.9)	4003 (47.07)	365 (46.92)	
Vigorous (≥ 45)	1954 (22.97)	128 (16.45)	
Hypertension, n (%)	1233 (14.50)	255 (32.78)	< 0.001
Dyslipidemia, n (%)	3659 (43.02)	520 (66.84)	< 0.001
Cardiovascular diseases, n (%)	1253 (14.73)	336 (43.19)	< 0.001
Abdominal obesity, n (%)	4601 (54.10)	525 (67.48)	< 0.001
General obesity, n (%)	2186 (25.70)	287 (36.89)	< 0.001
Fasting blood sugar (mg/dl)	90.45 ± 9.73	167.67 ± 61.56	< 0.001
Low-density lipoprotein cholesterol (mg/dl)	111.98 ± 30.97	110.56 ± 34.97	0.113
High-density lipoprotein cholesterol (mg/dl)	46.38 ± 11.24	43.77 ± 10.98	< 0.001
Triglycerides (mg/dl)	134.65 ± 77.33	182.97 ± 131.53	< 0.001
Total cholesterol (mg/dl)	185.23 ± 37.14	190.67 ± 43.85	0.001
Energy intake (kcal/day)	2723.62 ± 960.14	2592.36 ± 36.66	0.003

*T2DM *Type 2 diabetes mellitus, Continuous data are presented as mean ± SD and categorical data as number (%). The comparison between groups was done using chi^2^ test and independent t-test

### Logistic regression analysis of obesity and hypertension

Table 2 presents the independent association between obesity and hypertension on T2DM. Logistic regression analysis showed that abdominal obesity increases the odds of T2DM by 76% (OR: 1.76, 95% CI: 1.51, 2.10) and general obesity by 65% (OR: 1.65, 95% CI: 1.40, 1.94). After adjusting for confounding variables, this association was still significant. In the multivariate logistic model, the odds of T2DM in hypertensive participants was significantly higher by 84% (OR: 1.84, 95% CI: 1.55, 2.19).

**Table 2:** The relationship between general, abdominal obesity and hypertension with type 2 diabetes mellitus using logistic regression model

Parameters	Univariate^a^	*P* value	Multiple^b^	*P* value
	OR (95% CI)		OR (95% CI)	
Abdominal obesity				
No	Ref. (1.00)		Ref. (1.00)	
Yes	1.76 (1.51, 2.10)	< 0.001	1.58 (1.30, 1.93)	< 0.001
**General obesity**				
No	Ref. (1.00)		Ref. (1.00)	
Yes	1.69 (1.44, 1.97)	< 0.001	1.55 (1.31, 1.83)	< 0.001
**Hypertension**				
No	Ref. (1.00)		Ref. (1.00)	
Yes	2.87 (2.44, 3.38)	< 0.001	1.08 (1.01, 1.20)	< 0.001

*OR *Odds ratio, *CI *Confidence interval

^a^unadjusted

^b^adjusted for gender, age, residency, socioeconomic status, physical activity, smoking, cardiovascular diseases, and dyslipidemia

### Additive interaction analysis of general/abdominal obesity and hypertension on T2DM

Table 3 presents the odds of developing T2DM based on the different combinations of hypertension and general or abdominal obesity compared with the reference group (without both obesity and hypertension) by gender. Additive interaction analysis of abdominal obesity and hypertension on developing of T2DM in men shows, hypertension alone significantly increases the odds of T2DM by 1.66 times (95% CI: 1.18, 2.35) after adjusting for confounding variables. Abdominal obesity significantly increases the odds of T2DM by 1.51 times (95% CI: 1.14, 1.98). The combination of hypertension and abdominal obesity significantly increases the odds of T2DM by 2.38 times (95% CI: 1.67, 3.41). Similarly, in women, the highest risk of T2DM was in the combination of hypertension and abdominal obesity (OR: 4.02, 95%CI: 2.47, 6.47).

**Table 3:** Additive interaction of hypertension and obesity on the risk of type 2 diabetes mellitus by gender

Parameters 1	Parameters 2	Male		Female	
		Univariate^a^	Multiple^b^	Univariate^a^	Multiple^b^
		OR (95% CI)	OR (95% CI)	OR (95% CI)	OR (95% CI)
**Hypertension**	**Abdominal obesity**				
No	No	Ref. (1.00)	Ref. (1.00)	Ref. (1.00)	Ref. (1.00)
No	Yes	1.87 (1.20, 2.92)	1.50 (1.14, 1.98)	2.51 (1.61, 3.91)	2.12 (1.35, 3.34)
Yes	No	2.62 (1.19, 5.70)	1.66 (1.18, 2.35)	3.62 (1.65, 7.94)	2.24 (1.02, 4.98)
Yes	Yes	4.66 (2.93, 7.41)	2.38 (1.67, 3.41)	6.89 (4.34, 10.95)	4.02 (2.47, 6.47)
***Interactive effect***		*RERI: 1.17 (-0.76, 3.11)* *AP: 0.25 (-0.15, 0.65)* *SI: 1.47 (0.68, 3.17)*	*RERI: 0.27 (-1.01, 1.54)* *AP: 0.11 (-0.41, 0.63)* *SI: 1.23 (0.41, 3.68)*	*RERI: 1.74 (-0.99, 4.48)* *AP: 0.25 (-0.13, 0.63)* *SI: 1.42 (0.75, 2.68)*	*RERI: 0.61 (-1.12, 2.33)* *AP: 0.23 (0.08, 0.37)* *SI: 1.26 (0.60, 2.61)*
**Hypertension**	**General obesity**				
No	No	Ref. (1.00)	Ref. (1.00)	Ref. (1.00)	Ref. (1.00)
No	Yes	1.55 (1.21, 1.99)	1.43 (1.04, 1.97)	1.79 (1.39, 2.29)	1.81 (1.40, 2.36)
Yes	No	2.64 (1.98, 3.55)	1.58 (1.17, 2.15)	3.35 (2.49, 4.51)	2.11 (1.53, 2.91)
Yes	Yes	4.43 (3.23, 6.10)	2.53 (1.63, 3.82)	4.18 (3.04, 5.73)	2.94 (2.10, 4.05)
***Interactive effect***		*RERI: 1.25 (-0.11, 2.60)* *AP: 0.28 (0.03, 0.52)* *SI: 1.56 (0.97, 2.52)*	*RERI: 0.62 (-0.26, 1.49)* *AP: 0.23 (-0.05, 0.52)* *SI: 1.59 (0.81, 3.12)*	*RERI: 0.04 (-1.37, 1.44)* *AP: 0.01 (-0.32, 0.34)* *SI: 1.02 (0.64, 1.58)*	*RERI: -0.06 (-1.10, 0.96)* *AP: -0.02 (-0.38, 0.34)* *SI: 1.02 (0.56, 1.66)*

*OR *Odds ratio, *CI *Confidence interval, *RERI *Relative excess risk due to interaction, *AP *Attribution proportion, *SI *Synergy index

*unadjusted

**adjusted for residency, socioeconomic status, physical activity, dyslipidemia, age, smoking, alcohol drinking, energy intake, and cardiovascular

Additive interaction analysis of general obesity and hypertension on T2DM in women shows, after adjusting for confounding variables hypertension alone significantly increases the odds of T2DM by 1.58 times (95% CI: 1.17, 2.15). General obesity increases the odds of T2DM by 1.43 times (95% CI: 1.04, 1.97). The combination of hypertension and general obesity significantly increases the odds of T2DM by 2.53 times (95% CI: 1.63, 3.82). Similarly, in men, the highest risk of T2DM was in the combination of hypertension and general obesity (OR:2.66, 95%CI: 1.92, 3.70).

**Fig. 1:**
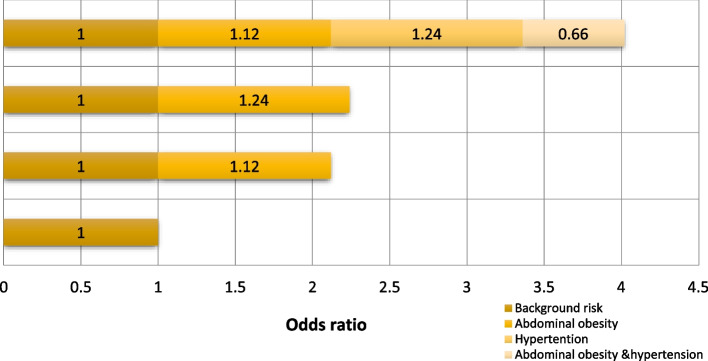
Additive interaction of hypertension and abdominal obesity on the risk of type 2 diabetes mellitus in male

**Fig. 2:**
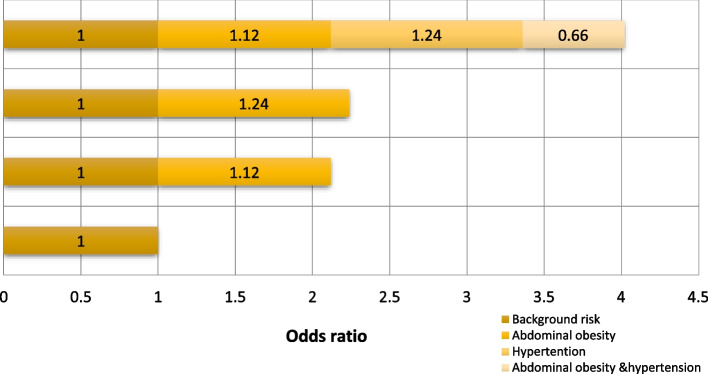
Additive interaction of hypertension and abdominal obesity on the risk of type 2 diabetes mellitus in female

In all cases of T2DM, 11% were attributed to the synergistic effect of abdominal obesity and hypertension (AP: 0.11), and the prevalence rate caused by the combination of the two factors was 0.27 times that caused by each factor independently (RERI). Moreover, 23% were attributed to the synergistic effect of general obesity and hypertension (SI: 0.23) in male. The study found that in women, abdominal obesity and high blood pressure had a significant synergistic effect on the development of T2DM (SI = 1.26). Figures 1 and 2 shows the interaction between hypertension and general/abdominal obesity in male and female.

## Discussion

This research demonstrates that the proportion of hypertension, general and abdominal obesity is higher in diabetics than in non-diabetics. The interaction analysis results revealed there is a synergistic effect between blood pressure and obesity (general/abdominal) in the development of T2DM, and this synergistic effect remained significant after controlling for confounding factors. Briefly, when obesity and hypertension coexist, the risk of T2DM is greater than the sum of obesity and hypertension alone.

The risk of T2DM in those with general/abdominal obesity was 1.65 and 1.69 times greater than in people without general/abdominal obesity, respectively after controlling for confounders. According to some studies [9, 19–21] obesity is a risk factor for diabetes, which accords with our results. In people with general or abdominal obesity, the accumulation of body fat causes hyperinsulinemia and insulin resistance, which reduces glucose tolerance and the development of T2DM by impairing the use of glucose by muscle and other tissues [9, 19], and obese people have a greater risk of acquiring diabetes than non-obese people do [9]. Losing weight is a crucial first step in lowering occurrence of T2DM, particularly in youthful people, as Studies have demonstrated that more than 80% of the physiological dysfunction of metabolic syndrome is caused by obesity [22]. Therefore, a person who is obese or overweight has a higher likelihood of experiencing diabetic remission the more weight they lose [23]. The association between abdominal obesity and elevated risk of diabetes was significant in the study population; and in women, the association was higher than in men. The higher prevalence of obesity among women can be used to explain this fact. Furthermore, lower levels of physical activity in females than in males, weight increase during pregnancy, and failure to recover to an ideal weight may be contributing factors to the greater occurrence of obesity and incident diabetes in females [20]. In accordance with our study, Wang et al. concluded that BMI-defined general obesity or WHpR-defined central obesity may be contributing factors for females with diabetes and hypertension. The simultaneous presence of hypertension with elevated BMI, WC, WHtR, and WHpR showed associations with the highest risks of developing diabetes [24].

The association between hypertension and T2DM was significant and there was 1.5 times increased risk of T2DM in hypertensive individuals compared to non-hypertensive individuals, which is consistent with a previous study [9, 25, 26]. It shows the importance of managing blood pressure and BMI effectively to prevent the onset and progression of diabetes [9].

However, there are limited finding on the interaction between general/abdominal obesity, hypertension, and the risk of T2DM. The findings of this research showed that the interactive effect of hypertension and abdominal obesity in men and women was 2.46 and 3.97 times the risk of T2DM. Interactive effect of hypertension and general obesity in men and women was 2.66 and 2.87, respectively. This result is in line with the findings from investigations by Conghui Hu et al. [9]. Several studies have investigated the interaction effects of two risk factors in increasing of risk of disease and have found similar results. Previous research has found that a family history of dyslipidemia and diabetes [27], a history of diabetes and high blood pressure in the family [28], and Having a family history of diabetes, as well as the waist-to-height ratio all work synergistically to impact the development of diabetes [29, 30]. In another study, it was shown that family history and overweight had a synergistic relationship with the pathogenesis of diabetes and that the impacts of the two factors independently were not insignificant [29]. When hyperlipidemia and familial history of diabetes coexist in normotensive populations, there may potentially be a synergistic influence on diabetes [9]. Studies like the ones described above have demonstrated that conditions like dyslipidemia, high blood pressure, and family history may increase or decrease a person’s chance of developing diabetes and that the cumulative incidence of illnesses has a higher effect on diabetes than the harm caused by a single complicating condition.

Obesity may impact blood pressure through various mechanisms, including leptin-mediated increased sympathetic activity and activation of the renin-angiotensin system. Additionally, insulin resistance is associated with greater sodium retention and increased blood pressure on a high-sodium diet [31]. The link between hypertension and T2DM can be explained by factors such as elevated blood sugar levels, insulin resistance, and dyslipidemia, all of which contribute to the development of atherosclerosis—a condition that can lead to vascular stenosis and heightened peripheral arterial resistance, both characteristic features of hypertension [32].

The interaction effect between obesity and hypertension on diabetes has important implications for clinical practice, as it indicates that these conditions should not be considered in isolation but rather as interrelated components of the metabolic syndrome. Therefore, it is necessary to implement comprehensive strategies to prevent and treat obesity, blood pressure, and diabetes, and to reduce the complications and deaths caused by them. Evidence-based strategies include lifestyle modifications such as weight loss, physical activity, dietary intervention, as well as pharmacological therapy [12].

The relationship between the two risk factors of obesity and hypertension on T2DM in a sizable group of Iranian people is being examined for the first time in this study. In our study, potential confounding variables (except genetic status) were controlled. The present study has a number of limitations, one of which is that it is cross-sectional in design and cannot demonstrate a causal association between risk factors and disease. Therefore, longitudinal studies are recommended to confirm the findings of this study.

## Conclusion

We found that general/abdominal obesity and hypertension have a synergistic effect on the risk of T2DM. To prevent the increasing incidence of T2DM, preventive strategies should be created that focus on these modifiable lifestyle factors. In order to avoid the occurrence and progression of T2DM, it is advised that people manage their hypertension and BMI through strategies like sensible exercise, a healthy diet, quitting smoking, and abstaining from alcohol.

## Data Availability

No datasets were generated or analysed during the current study.

## Abbreviations

T2DM: Type 2 Diabetes Mellitus
NCDs: Non-communicable Diseases
RaNCD: Ravansar Non-Communicable Disease
WC: Waist Circumference
BMI: Body Mass Index
SBP: Systolic Blood Pressure
DBP: Diastolic Blood Pressure
FBS: Fasting Blood Sugar
NCEP: National Cholesterol Education Program
PCA: Principal Component Analysis
MET: Metabolic Equivalent of Task
RERI: Relative Excess Risk Due to Interaction
AP: Attributable Proportion due to Interaction
SI: Synergy Index
OR: Odds ratio
CI: Confidence interval
